# Perceptions of Smoking and Vaping on Weight Control Among Adult American Indians Who Smoke

**DOI:** 10.1007/s10900-019-00694-x

**Published:** 2019-07-04

**Authors:** Dorothy A. Rhoades, Ashley L. Comiford, Justin D. Dvorak, Kai Ding, Michelle Hopkins, Paul Spicer, Theodore L. Wagener, Mark P. Doescher

**Affiliations:** 1 Stephenson Cancer Center, and University of Oklahoma Health Sciences Center, 655 Research Parkway, Suite 400, Oklahoma City, OK 73104 USA; 2 Epidemiology, Cherokee Nation, 1296 Skills Center Circle, Tahlequah, OK 74464 USA; 3 Hudson College of Public Health, University of Oklahoma Health Sciences Center, 801 NE 13th Street, Oklahoma City, OK 73104 USA; 4 Center for Applied Social Research, University of Oklahoma, 5 Partners Place, 201 Stephenson Parkway, STE 4100, Norman, OK 73072 USA; 5 Center for Tobacco Research, The Ohio State University Comprehensive Cancer Center, 460 W. 10th Ave, Columbus, OH 43210 USA

**Keywords:** Indians, North American, Electronic nicotine delivery systems, Vaping, Cigarette smoking, Body weight changes, Attitude, Adult

## Abstract

Interest in electronic cigarette (EC) use, or vaping, to help control weight is increasing. Many American Indian (AI) populations have a high prevalence of smoking, obesity, and EC use, but their perceptions of EC use for weight control are unknown. In Oklahoma in 2016, 375 AI adults who smoke completed a survey including perceptions about smoking and EC effects on weight control. Only 24% believed that smoking helps control weight, and 8% believed that vaping helps control weight. Perceptions differed by EC use, with ever users more often than never users perceiving that smoking (30% vs 12%, respectively; *p *< .01) and vaping (10% vs 5%; *p *= .04) help to control weight. Sex, age group (18–44 years vs 45 + years), education (high school graduate/equivalent vs less than high school), smoking cessation attempt in past year, and likelihood to quit in 6 months were not associated with weight control perceptions for either smoking or vaping. Uncertainty regarding EC effects on weight control was less common among EC ever users compared to never users (41% vs 53%, respectively; *p *= .04). Most people who did not believe or were uncertain that smoking controls weight also did not believe or were uncertain that vaping controls weight. However, only a minority (29%) of people who believed smoking controls weight also believed that vaping controls weight. Among adult AI who smoke, both smoking and vaping were infrequently perceived as helping to control weight, but such perceptions were reported more frequently among those who ever used ECs.

## Introduction

Smoking cessation is often associated with weight gain [1], and concerns about weight gain may affect smokers’ ability or desire to quit smoking. Such concerns often vary by race [2–5], sex [3, 5, 6], and body weight [3, 6]. Electronic cigarette (EC) use, or vaping, is increasingly prevalent among smokers, often as an aid for smoking cessation [7]. Evidence is also emerging that some people use ECs to control their weight [8, 9], and some researchers are beginning to express interest in using ECs to combat the obesity epidemic [10, 11], especially as obesity-related cancer incidence is on the rise [12].

While limited, some studies suggest that ECs may reduce the weight gain associated with smoking cessation. In a study of ex-smokers, those who did not use ECs gained more weight than persons who switched to ECs (4.8% of baseline weight vs 1.5%, respectively) [13]. In a study of smokers with hypertension, weight gain was higher among persons who quit smoking by switching completely to ECs (3.2 kg) compared with dual users (1.2 kg) or persons who continued to smoke (0.7 kg) [14]. However, these results compare favorably with 5–6 kg of weight gained historically at 12 months among adults who completely stop smoking [15–17].

Few studies examine perceptions of EC use regarding weight control among adults who smoke. In one study, obese persons were more likely to use cigarettes and/or ECs than non-obese persons [18], but perceptions of EC as helping to control weight were unreported. In another study, dual EC and cigarette users perceived that smoking provides better appetite and weight control than vaping, and women reported stronger appetite or weight control from vaping than did men [8].

American Indian (AI) adults in general have among the highest prevalence in the U.S. of smoking [19, 20], EC use [21, 22], and obesity [23, 24], but no studies explore perceptions of EC use for weight control among AI who smoke. Given that concerns about weight gain may affect the desire and ability to quit smoking, it is important to understand the perception of EC use on weight gain among AI who smoke. This report describes perceptions of the effects of smoking and EC use on weight control among a cohort of adult AI who smoke.

## Methods

Methods regarding study design, setting, recruitment, and select measures have been previously reported [25, 26]. Methods pertinent to the current analyses are provided briefly below.

### Study Design

Cross sectional baseline survey of a cohort of 375 adult AI men and women who smoke.

### Setting

Recruitment took place in 2016 within the Cherokee Nation Health Services (CNHS) primary care outpatient facility near tribal headquarters in Tahlequah, OK [25]. Nearly 190,000 Cherokee citizens reside in the Cherokee Nation Tribal Jurisdictional Service Area, which encompasses 14 predominantly rural counties in northeastern Oklahoma. The CNHS is the largest tribally operated healthcare system in the U.S. Patients are eligible for clinical services at CNHS if they show proof of AI descent, including a Certificate of Degree of American Indian or Alaska Native Blood (CDIB) from a federally recognized tribe. While Cherokee Nation citizens comprise the largest group of patients seen within the CNHS, patients from more than 300 tribes have utilized CNHS facilities.

### Participants

Study staff maintained an information table in a high-traffic waiting area within the outpatient facility. Signs and handbills announced the study and invited potentially eligible persons to participate. While most potential participants approached the information table first, staff also approached potential participants to provide information about the study. Due to the high traffic area, the total number of potentially eligible persons could not be determined.

Eligibility criteria for inclusion in the study included being age 18 years or older, smoking at least 100 cigarettes in lifetime, having smoked in the last 30 days, and answering, “Yes” to both “Are you American Indian?” and “Do you have a CDIB card?” Compensation for participation included a $20 gift card.

### Measures

*Perceptions of effects on weight control* by smoking or vaping were asked with two questions: “Do you think smoking helps people to keep their weight down?” and “Do you think vaping or using e-cigs helps people to keep their weight down?” Responses for each question included, “Yes”, “No”, and “Don’t know/Not sure”.

### EC Use

Pictorial examples of EC products were provided to help respondents recognize EC products [25, 27]. All participants were asked, “Have you ever vaped or used an e-cig even one or two times?” Individuals were considered never users if they answered “No” to this question. Persons who answered “Yes” were considered ever users.

*Demographic variables* included sex, age group (18–44 years vs 45 years and older), and education (less than high school graduate vs high school graduate).

Quit attempts in the past 12 months was assessed (yes/no) by the question “During the *past 12* *months*, have you stopped smoking for one day or longer because you were trying to quit?” Self-reported likelihood to quit in next 30 days was assessed with the question “How soon are you likely to quit smoking? Would you say…within the next 30 days, within the next 6 months, within a year, more than a year, or I am not likely to quit smoking”.

### Statistical Analysis

Descriptive data are shown as count (percent). Chi squared tests for independence were used to assess significant differences between groups in perceptions of smoking and vaping on weight, with Fisher’s exact test used for expected cell counts less than 5. Associations between smoking and vaping weight-control beliefs were examined using Chi squared tests for independence or, if warranted, Fisher’s exact test. If a significant association was observed for variables other than ever vaping, a logistic regression model was performed that incorporated history of ever vaping as an adjustment term. Missing data represented less than 3% of any of the variables studied. No data were imputed.

All statistical testing used SAS v9.4, SAS Institute Inc., Cary, NC, USA. A *p* value of 0.05 (two-sided) was the threshold for statistical significance.

## Results

Table 1 shows that only one quarter (24%) of participants believed that smoking helps keep weight down, while almost half (47%) believed smoking does not, and nearly one-third (29%) did not know or were unsure. Regarding ECs, less than 10% of participants perceived that ECs help keep weight down, nearly half (47%) did not, and nearly half (45%) did not know or were unsure.

**Table 1:** Perception of smoking and vaping on weight by vaping status, demographics, and smoking quit attempts among American Indians who smoke

	Smoking keeps weight down					Vaping keeps weight down				
	N	Yes [N (%)]	No ][N (%)]	DK/NS [N (%)]	*P* value	N	Yes [N (%)]	No [N (%)]	DK/NS [N (%)]	*P* value
Total	375	87 (24%)	174 (47%)	107 (29%)	–	364	30 (8%)	171 (47%)	163 (45%)	–
Vaping status					**< .01**					**.04**
Never	131	16 (12%)	73 (56%)	42 (32%)		129	6 (5%)	55 (43%)	68 (53%)	
Ever	234	71 (30%)	98 (42%)	65 (28%)		232	24 (10%)	113 (49%)	95 (41%)	
Sex					.49					.71
Men	138	28 (20%)	69 (50%)	41 (30%)		136	13 (10%)	61 (45%)	62 (46%)	
Women	230	59 (26%)	105 (46%)	66 (29%)		228	17 (7%)	110 (48%)	101 (44%)	
Age group, years					.74					.13
18–44	211	53 (25%)	98 (46%)	60 (28%)		208	21 (10%)	102 (49%)	85 (41%)	
45 +	157	34 (22%)	76 (48%)	47 (30%)		156	9 (6%)	69 (44%)	78 (50%)	
Education					.41					.91
< HS graduate	74	13 (18%)	38 (51%)	23 (31%)		74	7 (9%)	34 (46%)	33 (45%)	
HS graduate	293	73 (25%)	136 (46%)	84 (29%)		289	23 (8%)	137 (47%)	129 (45%)	
Quit attempt past 12 month					.37					.05*
Yes	144	39 (27%)	67 (47%)	37 (26%)		142	17 (12%)	70 (49%)	55 (39%)	
No	227	48 (22%)	104 (47%)	69 (31%)		219	13 (6%)	99 (45%)	107 (49%)	
Likely to quit in 30 days					.14					.73
Yes	45	13 (30%)	24 (55%)	7 (16%)		43	3 (7%)	23 (54%)	17 (40%)	
No	326	74 (23%)	149 (47%)	97 (30%)		317	27 (9%)	147 (46%)	143 (45%)	

Percentages reflect row totals, and may not equal 100 due to rounding

*After adjustment for ever vaping, the association became non-significant (*p *= 0.10)

*DK/NS* don’t know/not sure, *HS* high school

Perceptions that either smoking or vaping helps to keep weight down varied significantly by vaping status but not by sex, age group, or education. Ever vapers more often than never vapers reported the belief that smoking helps keep weight down (30% vs. 12%, respectively; *p *< 0.01). Overall, ever vapers also differed from never vapers in perception of EC effects on weight (*p* = .04). Compared with never vapers, ever vapers more often believed that vaping helps keep weight down (10% vs 5%) and less often expressed uncertainty about the effect of ECs on weight (41% vs 53%). When assessing the proportions expressing uncertainty (don’t know/not sure) versus certainty (yes and no) about EC effects on weight, the differences between ever vaper and never vapers remained significant (*p *= .04).

No significant association occurred between attempt to quit smoking in the past 12 months and perception of smoking as controlling weight. Although the association between past 12 months quit attempt and perception of vaping as controlling weight was significant in the univariate analysis (*p *=0.05), it was not significant (*p* = 0.10) after adjustment for ever vaping. Neither weight-control belief variable was significantly associated with self-reported likelihood to quit in the next 30 days.

Table 2 shows a significant association between weight-control beliefs overall. Within groups, most (141/172; 82%) participants who did not believe smoking helps control weight did not believe that vaping helps control weight. Most (100/106; 92%) persons uncertain about smoking effects on weight were also uncertain about EC effects on weight. However, among participants who believed that smoking helps keep weight down, only 29% (25/86) also believed that vaping keeps weight down, 29% (25/86) did not believe that vaping keeps weight down, and 42% (36/86) were unsure.

**Table 2:** Association between perceptions of weight control beliefs for smoking and EC use among American Indians who smoke

	EC use helps control weight		
	Yes	No	DK/NS
Smoking helps control weight			
Yes	25 (7%)	25 (7%)	36 (10%)
No	4 (1%)	141 (39%)	27 (7%)
DK/NS	1 (0.3%)	5 (1%)	100 (27%)

Chi squared test for independence = 241.23; *p* < .0001

Percentages add to 100; N missing = 11

*EC* electronic cigarette, *DK/NS* don’t know/not sure

## Discussion

In this study of adult AI who smoke, only a minority perceived that either smoking or vaping helps people to keep their weight down. In contrast, smoking and increasingly vaping are perceived as helping to keep weight down among many non-AI smokers in the U.S. [10, 28–33]. In one US study, people who smoke perceived that cigarettes were better for weight control than were ECs [34]. Among non-AI smokers with psychological distress, ECs were perceived as having favorable effects on weight control, although less so than cigarettes [33]. In a study of young adults, weight concerns were associated with past 30 day EC use [32].

Weight concerns among AI who smoke may differ from the general population. In one study, AI had a lower expectation than Whites did that smoking cessation would cause weight gain [5]. In another, concerns regarding weight were less commonly reported by AI women than by women from other racial groups [3]. Among adolescent girls, AI did not differ from Whites in smoking for weight control, but among adolescent boys, AI more often smoked to control weight than did Whites [35]. The low proportion of persons in the study endorsing the perception that smoking or vaping help keep weight down suggests that weight control was not a major impetus for using or continuing to use either of these products.

Although only a small proportion of AIs in this study perceived either product as a way to keep weight down, a history of ever vaping was slightly associated with a higher frequency of perceiving ECs as helping to keep weight down. One study similarly found that vapers more often than non-vapers believed that vaping helped control appetite and weight [36]. Whether AI who use ECs do so in part as a way to keep weight down needs further research, as do the implications such use may have for smoking cessation and overall EC use.

No significant association occurred by sex, age group, or education regarding weight control perceptions for either smoking or vaping. Although a higher proportion of women than men believed smoking helps keep weight down, the difference was not significant. The differences between men and women were even less for vaping effects on weight. Other studies vary regarding the effect of sex on perceptions of EC and weight control among adults. In a study of vapers, 13.5% vaped to lose weight or to control weight, but neither smoking nor sex was associated with such use [8]. In contrast, other studies of EC users found that female participants rated ECs higher for weight control than did men [36–38]. Age was not significantly associated with vaping for weight loss in another study of EC users [8], but age has not been reported in association with perceptions of weight control in other EC studies of adults. However, weight concerns associated with smoking cessation may be more prominent among younger smokers than among older smokers [4, 39]. A study was not found that assessed education in association with perceived effects of ECs on weight control in other studies of adults in the U.S.

Even though a slight association occurred between quit attempt and belief that vaping helps keep weight down, the proportion of people who both tried to quit and believed that vaping helps control weight was very small. Although the study did not directly assess whether people vaped or smoked in order to help control their weight, these data do not suggest that AI who smoke are frequently motivated to use ECs for weight control purposes. The impact of weight control concerns on smoking cessation among this population deserves greater study, as does directly inquiring whether AI people who smoke use ECs to reduce the weight gain that accompanies smoking cessation.

Unique to this study is the assessment of the prevalence of uncertainty regarding the effect of smoking and vaping on weight control. Because ECs are new and rapidly evolving products, and debate exists among public health researchers about their harms and benefits [40, 41], uncertainty regarding their effects is expected. The prevalence of uncertainty regarding vaping’s effects on weight control overall was high with nearly one-half of the cohort responding, “don’t know/not sure.” The degree of uncertainty differed significantly by vaping status, with ever vapers reporting significantly less uncertainty than the never users, but not by sex, age group, or education. This may be due to ever vapers being more familiar with EC products than never vapers.

Only a minority of people who believed that smoking helps control weight also believed that vaping helps control weight. Again, this suggests that weight control beliefs were not common motivators for vaping.

The relative benefits versus harms of switching to vaping among adult smokers remain to be seen. Nicotine regulates feeding and metabolism by complex, multilevel actions that result in both increased energy expenditure and reduced food intake [42]. Whether these effects of nicotine are preserved among persons who vape has yet to be determined. The potential for vaping to help control weight is of increasing interest [10], and is of particular importance among AI populations with high a prevalence of obesity and smoking. However, in this sample of AI who smoked, most respondents, even those who also vaped, did not perceive that ECs help control weight. Switching from cigarettes to ECs may result in less weight gain than quitting without using ECs [13], but such effects require prospective study among AI populations [43]. Among Alaska Natives, but not among AI, genetic variants have been associated with an interaction between nicotine intake and higher body mass index [44, 45], but whether this holds true for electronic nicotine sources has not been studied [45].

### Limitations

Small sample size and convenience sampling limit this study, which could not detect modest associations. Other studies suggest that being overweight is associated with use of ECs among adults [8, 18, 38], and EC use has been associated with eating disorders [9], but the present study did not assess the body mass index of participants.

## Conclusion

Although differing somewhat by vaping status, the perception that EC use helps to control weight was uncommon, and uncertainty was common, among adult AI who smoke. Future studies should directly assess whether weight concerns influence the use of ECs among AI, whether such use actually affects their weight, and whether such weight concerns affect their smoking cessation.

## Data Availability

Restrictions apply to the availability of these data, which were used under agreement with Cherokee Nation, and are not publicly available. Data are, however, available with explicit permission of Cherokee Nation.
